# Bone Marrow Micrometastases in Breast Cancer Patients: A Long-Term Follow-up Study

**Published:** 2008-09-04

**Authors:** Annamaria Molino, Monica Giovannini, Rocco Micciolo, Alessandra Auriemma, Elena Fiorio, Antonio Santo, Gian Luigi Cetto

**Affiliations:** 1 Department of Medical Oncology, University of Verona, Verona, Italy; 2 Department of Medical Oncology, San Raffaele Hospital, Milan, Italy; 3 Institute of Statistics, University of Trento, Trento, Italy

**Keywords:** bone marrow, breast cancer, long-term follow-up, micrometastases

## Abstract

In 125 early breast cancer patients who underwent multiple bone marrow aspirates, there was no significant difference in terms of disease-free and overall survival after a median follow-up of 163 months between the patients with or without micrometastasis at the time of primary surgery. However, when the time-dependent evolution of the bone marrow aspirates was taken into account, some evidence for a longer disease-free and overall survival was found for the patients with negative bone marrow

## Introduction

Epithelial cells in bone marrow may be a prognostic factor in patients with primary breast cancer (BC). We have previously found that the evolution of bone marrow micrometastases in a series of women with primary breast cancer diagnosed between 1990 and 1994 did not correlate with treatment or non-treatment [1,2], or prognosis. We here update the findings after a median follow-up of 163 months of women who underwent bone marrow analysis at the time of surgery and every 6–8 months afterwards.

## Patients and Methods

Between January 1990 and December 1993, 125 patients with operable (stage I and II) breast cancer admitted to the Department of Medical Oncology of the University of Verona underwent bone marrow aspiration at the time of surgery, called Time 0, and repeated in respect of patient compliance every 6–8 months, starting at the end of adjuvant chemotherapy or at the first 6–8 month follow up time. The bone marrow preparation procedure has been previously described [2]. The samples were processed for leukocyte separation, used to prepare cytospin slides, stained with a pool of monoclonal antibodies (including MBr1, MBr8, MOV8, MOV16 and MluCI, provided by Dr. M.I. Colnaghi, Istituto Nazionale Tumori, Milan, Italy). We here describe the 13-year follow-up analysis with the estimated disease-free (DFS) and overal survival (OS).

## Data Analysis

The probabilities of DFS and OS were estimated using the product-limit method [3]. The effect of micrometastases on relapse-free and overall survival was evaluated using Cox’s proportional hazards model [4]. The significance tests were based on the likelihood ratio statistic. The time-dependent evolution of bone marrow status as a possible prognostic factor was evaluated using the approach suggested by Andersen and Gill [5], who reformulated the proportional hazards model as a counting process.

## Results

The 125 women were followed up to 31/8/2005, for a median follow-up of 163 months. During the observation period, 54 patients (43.2%) relapsed and 45 (36.0%) died; the first bone marrow sample was positive in 17 of the relapsing patients (31.5%) and 15 of those who died (33.3%). Of the 71 non-relpsing patients and 80 survivors, respectively 22 (31.0%) and 24 (30.0%) had a positive first bone marrow sample.

The 10-year probability of relapse-free survival was quite similar in the patients with micrometastases (0.632; 90% C.I.: 0.515–0.774) and in those without (0.572; 90% C.I.: 0.488–0.670). Similar results were found for overall survival, where the 10-year survival probability was respectively 0.683 (90% C.I.: 0.569–0.820) and 0.674 (90% C.I.: 0.592–0.767). Cox’s model showed that the hazard for both relapse and death was not significantly different between patients with and without micrometastases at the time of surgery.

However, the result of a bone marrow aspirate can be considered a time-dependent variable, because at subsequent evaluations, a previously positive result can disappear or a shift from a negative to a positive result can be found. For example, in the 74 patients with two aspirates, a shift from a negative to a positive result (−/+) was found in 8 women, while a shift from a positive to a negative result (+/−)was found in 14 women. The exact binomial test for paired proportions yielded a *p* value of 0.286, thus indicating no significant difference in the probability of the −/+ and +/− patterns.

As a matter of fact, there were a total of 32 shifts in the 26 patients who had more than one aspirate. To account for the time-dependent evolution of the bone-marrow aspirate, the approach of Andersen and Gill [5] was employed. Briefly, as time marches onward, we observe the results for a subject; the data for a woman with one or more shifts are presented as multiple “observations”, each of which applies to an interval of her follow-up time.

After having taken into account the time-dependent evolution of the bone-marrow aspirates, the hazard ratio of relapse (positive *vs* negative bone marrow) was 1.87 (90% C.I.: 1.09–3.22), and that of death was 1.79 (90% C.I.: 1.05–3.05).

## Discussion

Metastasis, the spread of invasive carcinoma to sites distant from the primary tumor, is responsible for the majority of cancer-related deaths. Despite progress in other areas of cancer therapeutics, the complexities of this process remain poorly understood. The long prevailing model of metastasis recognizes the importance of both “seed” and “soil” for metastatic progression [6]. Much attention has been paid to the relationship between the presence of cancer cells in bone marrow and patient outcome. Most published papers report an association between bone marrow positivity and a poor prognosis in breast cancer patients [1,8–25], including a recent meta-analysis [25] that found a correlation with micrometastases in bone marrow at the time of diagnosis in 4703 patients after a median follow-up of 5.2 years. Even with a median follow-up of almost 14 years, we did not find this correlation when we only considered the aspirates taken at the time of surgery, i.e. the first aspirates, but found it almost reached statistical significance in terms of both DFS and OS when all of the aspirates were included. In other words there is the evidence that the bone marrow evolution over time, rather than bone marrow status at the time of surgery, seems to have a potential prognostic role. However on the other hand one reason for reaching no significance might be that many breast cancer cells were proved to be apoptotic and so without any consequences for the patient.

Our analysis is characterised by the particularly large number of multiple bone marrow aspirates, which were repeated, according to patient compliance, every 6–8 months starting from the time of surgery and the very long median follow-up (163 months). However we should consider that the possibility to repeat the bone marrow aspirates over time is invariably linked to the disease evolution. The results suggest that bone marrow micrometastases probably have a major impact on DFS and OS for at least 10 years after diagnosis, and that finding a second bone marrow aspirate positive is more informative than finding a positive first aspirate. We could speculate that probably a long lasting cross talk between “seed” and “soil” may be more significant in inducing the metastatic process.

However many factors could help to interpret our results on such an interesting and intriguing topic, still arising many questions.

Firstly a better understanding of the relationship between bone stromal components, cancer cells and hematopietic cells. In fact recently much attention has focused on understanding the molecular and genetic factors that confer an intrinsic metastatic advantage to certain tumor cells. Meanwhile, changes occurring within distant tissues, creating a “soil” conducive for tumor invasion, have been largely neglected. Recent work characterizing the importance of bone marrow-derived hematopoietic progenitor cells in initiating these early changes has opened new avenues for cancer research and chemotherapeutic targeting [26]. In fact bone marrow-derived hematopoietic progenitor cells recently emerged as key players in initiating these early changes, creating a receptive microenvironment at designated sites for distant tumor growth and establishing the “Pre-Metastatic Niche” [27]. This insight into the earliest stages in the metastatic cascade revises our concept of the metastatic “microenvironment” to include physiological cells recruited from the bone marrow. Moreover, the concept of pre-metastatic tissues as ‘niches’ similar to physiological stem cell niches establishes a paradigm in which disseminated tumor cells may reside within a highly defined microcosm, both supportive and regulatory, and which may confer specific functions on indwelling cells. Understanding the cellular and molecular cross-talk between “seed” and “soil” may further our understanding of the factors that govern both site-specific patterning in metastasis and the phenomenon of tumor dormancy. This may lead to therapeutic strategies to detect and prevent metastasis at its earliest inception.

Secondly a greater knowledge of the biologic factors of primary tumors could be helpful; in fact even if the prognostic significance of disseminated tumor cells in the bone marrow of breast cancer patients has been demonstrated in many studies, yet, it is not clear which of the primary tumors’ biological factors predict hematogenous dissemination [28] and it’s still under investigation.

Thirdly the technics used have also to be considered. Over the past 15 years early tumor cell dissemination has been detected in patients with breast cancer using sensitive immunocytochemical and molecular assays based on the use of MAb and PCR, respectively. Tumor cells have been detected either directly, using immunocytochemical staining, or indirectly, using reverse transcription-polymerase chain reaction (RT-PCR) as recently published [29–31]. These studies carried out in both primary and metastatic breast cancer patients described the methodologies and markers used, and improvements in detection methodologies that are being investigated including real-time RT-PCR, novel markers, enrichment and automated image analysis. Additionally advantages and limitations of the technics used to detect cancer cells have been analysed by many authors [30–33]. Advanced methods for molecular characterization of single tumor cells and the surrounding environment have been developed lately, and this approach allows new insights into the metastatic cascade and characterization of targets for therapeutic approaches.

Taken together all this observations lead to the conclusion that, even if started many years ago and using the technics available at that moment, our study, thanks to the very long follow up time and the high number of bone aspirates taken, has to be considered in interpreting the role of bone marrow micrometastasis in breast cancer patient long term outcome.

Further prospective studies are required to examine this possibility in greater detail, with particular reference to early node-negative breast cancer and the value of adjuvant systemic therapy in patients with bone marrow micrometastasis, above all after a long follow-up. Further studies are also needed to increase the sensitivity and reproducibility of detecting micrometastases by technologically advanced means such as PCR or by using different monoclonal antibodies.
